# The Diversity Compass: a clinical ethics support instrument for dialogues on diversity in healthcare organizations

**DOI:** 10.1186/s12910-023-00992-z

**Published:** 2024-01-03

**Authors:** Charlotte Kröger, Bert Molewijk, Maaike Muntinga, Suzanne Metselaar

**Affiliations:** 1 Department of Ethics, Law and Humanities, Amsterdam UMC Location Vrije Universiteit Amsterdam, De Boelelaan 1089a, Amsterdam, The Netherlands; 2 Netherlands Defense Academy, Breda, The Netherlands; 3 Centre for Medical Ethics, University of Oslo, Oslo, Norway

**Keywords:** Diversity, Inclusion, Social justice, Healthcare, Healthcare organizations, Clinical ethics support

## Abstract

**Background:**

Increasing social pluralism adds to the already existing variety of heterogeneous moral perspectives on good care, health, and quality of life. Pluralism in social identities is also connected to health and care disparities for minoritized patient (i.e. care receiver) populations, and to specific diversity-related moral challenges of healthcare professionals and organizations that aim to deliver diversity-responsive care in an inclusive work environment. Clinical ethics support (CES) services and instruments may help with adequately responding to these diversity-related moral challenges. However, although various CES instruments exist to support healthcare professionals with dealing well with morally challenging situations in healthcare, current tools do not address challenges specifically related to moral pluralism and intersectional aspects of diversity and social justice issues. This article describes the content and developmental process of a novel CES instrument called the Diversity Compass. This instrument was designed with and for healthcare professionals to dialogically address and reflect on moral challenges related to intersectional aspects of diversity and social justice issues that they experience in daily practice.

**Methods:**

We used a participatory development design to develop the Diversity Compass at a large long-term care organization in a major city in the Netherlands. Over a period of thirteen months, we conducted seven focus groups with healthcare professionals and peer-experts, carried out five expert interviews, and facilitated four meetings with a community of practice consisting of various healthcare professionals who developed and tested preliminary versions of the instrument throughout three cycles of iterative co-creation.

**Results:**

The Diversity Compass is a practical, dialogical CES instrument that is designed as a small booklet and includes an eight-step deliberation method, as well as a guideline with seven recommendations to support professionals with engaging in dialogue when they are confronted with diversity-related moral challenges. The seven recommendations are key components in working toward creating an inclusive and safe space for dialogue to occur.

**Conclusions:**

The Diversity Compass seeks to support healthcare professionals and organizations in their efforts to facilitate awareness, moral learning and joint reflection on moral challenges related to diversity and social justice issues. It is the first dialogical CES instrument that specifically acknowledges the role of social location in shaping moral perspectives or experiences with systemic injustices. However, to make healthcare more just, an instrument like the Diversity Compass is not enough on its own. In addition to the Diversity Compass, a systemic and structural approach to social justice issues in healthcare organizations is needed in order to foster a more inclusive, safe and diversity-responsive care and work environment in health care organizations.

**Supplementary Information:**

The online version contains supplementary material available at 10.1186/s12910-023-00992-z.

## Introduction

Increasing social pluralism is associated with growing variation in health beliefs, care practices, needs and communication preferences, and means that different moral perspectives exist among and between healthcare professionals and various patient (i.e., care-receiver) populations on what ‘good care’ entails [1–4]. These differences in moral positions can relate to various intersectional aspects of diversity, including a person’s upbringing, social background, class, ability, culture, race and ethnicity, religion or spirituality, sexual and gender identity [5]. This calls for care and organizational practices that are responsive to the different social locations of those receiving care [6, 7].

Healthcare professionals and organizations are increasingly confronted with moral challenges that are related to social justice issues, dealing with social inequalities and structural health disparities [3, 8–15], and to addressing heterogeneous moral perspectives on what it means to respond to various aspects of diversity in an inclusive, equitable and just way in care practice [1, 2, 4, 16]. Moral challenges are difficult situations where stakeholders’ values conflict or where stakeholders experience moral uncertainty about the ‘right’ course of action [17]. By using the term ‘moral’ challenge, we acknowledge that the challenges that individual healthcare professionals may experience in practice are often connected to their own moral compass and lived experiences as people and professionals, and to their personal understandings of what is good in a specific situation. While morality encompasses (moral) pluralism between people, ‘ethical’ challenges, to us, more broadly refer to formal, thematically abstract and shared ethical beliefs that exist within certain social systems. In this article, the term ‘diversity-related moral challenges’ refers to practical situations in healthcare, in which the moral issue at hand relates to social justice and is intertwined with differing values, moral positions, lived experiences and social locations of people with different (majoritized and minoritized) backgrounds, cultures, and identities.

Some examples of diversity-related moral challenges that healthcare professionals may encounter in practice are: ought a physician accommodate religion-related care preferences on administering morphine at the end of life when they are at odds with the protocol? How should a healthcare manager deal with patients that have clear gender preferences in who ought to care for them? What is the right thing to do when you and your colleagues have a different perspective on how to support a transgender patient who is transitioning? Or when you don’t feel accepted at work because of your cultural background or spiritual beliefs? And what is a good way to inquire about someone’s gender identity or sexuality without trespassing personal boundaries? The ability to morally reflect on and deal with such complex diversity-related questions in a good way is vital for healthcare professionals to carry out their everyday work, both individually and at team level, for their own well-being, but also for the quality of care they provide to different patient populations.

Diversity can be conceptualized in different ways [18]. In this paper, we define diversity as intersectional differences in identity that shape a person’s social location and experiences, and create different modes of discrimination and privilege [5] in healthcare settings [19, 20]. Diversity-responsiveness refers to being responsive toward different aspects of diversity and how these affect the way care services are accessed, delivered, received and promoted in policy, practice and care services [16, 18]. There remains a lack of awareness, knowledge, and competences among healthcare professionals regarding how they ought to deliver diversity-responsive care to all care-receivers [16]. Furthermore, professionals’ personal experiences and cultural backgrounds may lead to implicit blind-spots and biases toward (minoritized) others [21–23].

It has been argued that clinical ethicists can and should support healthcare professionals in becoming (more) diversity-responsive, and in promoting social justice and addressing racism in healthcare [24–26]. Rather than providing rules, advice or guidelines on what constitutes ‘the right thing’ to do, clinical ethicists may support professionals to methodically address their moral doubts, conflicts, and dilemmas in clinical practice through joint, dialogical reflection on concrete cases [27–30]. This approach to clinical ethics support (CES) is based on philosophical pragmatism and hermeneutics [2, 31, 32]. The main underlying idea is that fostering methodically structured dialogues can facilitate openness, mutual understanding, moral learning and moral competencies of healthcare professionals, patients and other relevant stakeholders [25, 28, 33].

Various dialogical CES services and instruments exist that provide guidance for healthcare professionals to jointly reflect on morally challenging situations in practice [27, 30, 31, 34–38]. However, these often overlook the role of social location in shaping moral values, perspectives on good care, or experiences with systemic injustices, and do not sufficiently include minoritized voices in the developmental process. A CES instrument that is developed with end-users and actively pays attention to diverse backgrounds and social identities in shaping moral perspectives does not yet exist.

The objective of this study was to develop a CES instrument to support healthcare professionals with addressing moral challenges related to intersectional aspects of diversity and social justice issues. We developed this instrument in a participatory way, i.e. working closely together with a diverse group of stakeholders. We have previously argued that it is crucial to facilitate an inclusive and participatory process when engaging in CES on diversity issues in healthcare organizations [25]. A participatory development design is key to develop an intervention that is responsive to the needs, perspectives and moral challenges of all end-users, thereby empowering them and increasing the chance for social impact to occur [36, 39, 40].

In this article, we present both the content and development of this instrument. First, we will elaborate on the participatory development (PD) process that led to the Diversity Compass. Then, we will present the two key elements of the instrument: (1) seven recommendations to support professionals when engaging in dialogue about diversity-related moral challenges, and (2) a deliberative method that consists of eight steps, in order to methodically structure this dialogue. We will also provide a short case example to illustrate the way in which the Diversity Compass can be used. In our discussion, we critically reflect on the content and development process of the Diversity Compass.

## Methods

### Research design

This study was conducted between 2019 and 2020 in a large long-term care organization in a major city in the Netherlands. We used a participatory development (PD) design [36, 41, 42]. PD is a form of action research that is based on close collaboration between researchers and participants. The underlying normative idea of participatory practices is that to develop meaningful, innovative and useful practical instruments that have social impact, end-users ought to be included in the developmental process [39]. The goal is to change (care) practice by developing solutions, instruments or services in response to practical problems with those that experience them [43].

This approach is well-suited for conducting research and developing instruments on diversity and social justice issues specifically, as PD pays specific attention to the values and wishes of relevant stakeholders, also those whose voices are usually not heard. This leads to a more just developmental process and also enhances the chance for social impact to occur, as the outcome is more responsive to the needs of the end-users [39, 40]. Additionally, PD is in line with our approach to CES that is rooted in philosophical pragmatism, discourse ethics and hermeneutic ethics and focusses on joint reflection with relevant stakeholders in order to facilitate moral learning on the basis of concrete experiences and contextual knowledge [2, 25, 28, 31–33, 36].

**Fig. 1:**
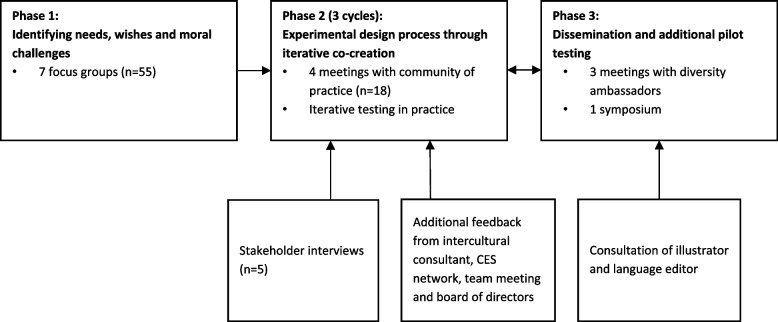
Overview of the participatory development process

While there are different approaches to PD, this study distinguishes three phases [41]. Phase one concerns identifying user needs prior to the development of an instrument. Phase two, the experimental design process, focuses on designing and developing the instrument through iterative co-creation [42]. Iterative co-creation is a cyclical process aimed at developing tailored solutions based on stakeholders’ needs and circumstances [44]. The iterative cycles consist of the elements planning, acting, observing and reflecting [43]. Phase 3 regards additional pilot testing and dissemination of the tool in practice. PD and participatory practices have been employed previously to develop ethics support instruments [36] and organizational diversity statements through dialogical ethics support [25]. An overview of the PD process that lasted 13 months is shown in Fig. 1. Because of the iterative nature of PD, each element of the process was planned based on ideas and outcomes generated in the previous activity.

### Participatory development process

In this section we elaborate on the PD process that led to the development of the Diversity Compass.

#### Phase 1

In phase one of the PD process, CK conducted seven focus groups to understand which diversity-related moral challenges professionals encountered and what their ideas, wishes and needs were regarding the development of a diversity-responsive ethics support tool. We employed the focus group method as an interactive and dialogical way to explore, reflect on and exchange viewpoints among participants [45]. This provided deeper insight into the complexities and variations of existing moral perspectives and experiences with diversity and social (in)justices in healthcare within the groups.

The long-term care organization where this study took place had established a network of ‘diversity ambassadors’: healthcare professionals who are voluntarily and actively involved in promoting diversity-responsiveness at different locations of the organization. At the time this study was conducted, this network consisted of four groups of professionals that met regularly to discuss concrete cases and ways of increasing inclusion and social justice in their daily practice. Alongside these meetings, their tasks include giving presentations, workshops and addressing injustices on the workfloor in different ways. The first four of the focus groups were held with four of these pre-existing groups of diversity ambassadors. Subsequently, using snowball sampling, three additional focus groups were conducted. All participants were initially contacted by email and agreed to participate in the focus groups.

Fifty-five people participated in the focus groups, three of which participated twice. The three participants that took part twice, did so as they were members of two different diversity ambassador groups. 15 participants were male and 38 participants female. They were between 29 and 65 years of age. All participants were Dutch nationals. More than one third of them identified as being from Moroccan, Surinamese, Antillean, Afghan, Indonesian or Indian descent, several had varying religious and spiritual beliefs, and about one in ten voluntarily offered information about identifying as LGBTQIA + . The participants worked at different branches of the long-term care organization, i.e. elderly care, mental health care and disability care. Six of the focus groups were heterogeneous in terms of professions, with participants being managers, spiritual counselors, nurses, activity coaches, intercultural consultants, doctors, healthcare counselors, members of HR, social workers and policy advisors. One of the seven focus groups was homogeneous and held with four patients who were peer-experts, three of which were also members of the patient advisory board. In the Netherlands, peer-experts are former and current patients who, after receiving a formal peer-support education themselves, educate and support healthcare professionals and patients on dealing well with various health and decision-making topics, based on their own experiences. Their perspectives provided additional insight into some of the moral challenges related to diversity and social justice issues, experienced from a patient perspective.

All focus groups were guided by a semi-structured topic list (see Supplementary file for the interview guide). Topics included questions about how respondents defined diversity, their personal experiences with moral challenges related to diversity and inclusion in their respective work settings and their wishes and needs regarding the development of an ethics support instrument in order to deal with diversity-related moral challenges in practice. Six focus groups were recorded, and detailed field notes were taken during the seventh focus group. The seventh focus group was not recorded due to an issue with the voice recorder.

The focus groups yielded important data on healthcare professionals’ and peer-experts’ experiences with diversity-related moral challenges and led to several concrete suggestions on what the ethics support instrument ought (not) be (see Table 1).

**Table 1:** Suggestions of focus group participants regarding the ethics support tool

Focus group participants made the following key suggestions regarding the tool
**The tool should:**
1) be low-threshold,
2) offer support on how to reflect on a moral issue by discerning values, naming emotions and suspending prejudices,
3) be directed at everyone, majoritized and minoritized healthcare professionals,
4) be applicable to different care environments, and
5) provide recommendations for social safety and ways to be open, honest and vulnerable
**The tool should not:**
1) be too lengthy or complex,
2) consist of normative rules or a checklist, and
3) reinforce prejudices

#### Phase 2

Taking the outcomes of the focus groups as a starting point, we established a working group, or *community of practice*. A community of practice refers to a group of people that “share and create relevant knowledge, skills, and best practices” through social interaction [46]. This group was facilitated by CK and SM and was a key element of the second phase of the PD design. Through sharing experiences, reflections and experimenting with preliminary versions of the instruments in practice, the participants of the community of practice jointly developed the Diversity Compass in a process of iterative co-creation (phase two) [44, 47].

CK, SM and a director of the healthcare organization who was also the diversity officer first deliberated on who ought to be included in the group. We chose for maximum variation in professions and social identity. Based on the directors’ network, we approached possible participants that also had affinity with the subject by mail, inquiring if they were interested in joining the community of practice. Eighteen stakeholders were approached, all of which agreed to participate. Ten were female and eight were male. They were between 29 and 64 years of age. All were Dutch nationals. About half of the participants were of different descent, and/or identifying with a specific religion and/or identifying as LGBTQIA + . Their professions were: managers at different levels of the organization, policy advisors, a spiritual counselor, nurse, intercultural consultant, doctor, psychiatrist and a peer-expert (i.e., former patient). They worked in elderly care, disability care or mental health care. In four meetings of two hours that took place in six-week intervals, the community of practice co-created the instrument by engaging in dialogue on the goal, content, layout and design. They also reflected on personal and concrete experiences with diversity, privilege, exclusion and social justice.

During the first session of the community of practice, we specifically deliberated on the outcome of the focus groups, recognized that the tool should be relevant to all (minoritized and majoritized) healthcare professionals for the organization and decided that it should facilitate and provide practical recommendations (from which we identified three) on how to engage in dialogue on diversity-related moral challenges. During the second meeting we reflected on the terms diversity and inclusion and on personal experiences of participants with a diversity-related moral challenge. Based on these experiences we identified further practical recommendations and agreed that the tool should also provide a structured deliberation method and concrete examples. In the third session we tried out the deliberation method by deliberating on a case within the group. Based on the ongoing dialogue, we altered, removed and added some questions as a result of this process and defined further recommendations for engaging in dialogue on diversity with each other. The outcome was the final deliberation method, comprising eight steps. In the fourth session, we discussed final considerations related to the content of the tool, ideas about the design and layout and what was necessary for a good implementation of the Diversity Compass. The group decided that the researchers should also try-out the instrument in other contexts within the organization to increase awareness of the Diversity Compass among other professionals and thereby enhance social impact and implementation.

### Interviews and additional feedback

We also consulted additional stakeholders between the meetings to obtain more feedback on preliminary versions of the Diversity Compass. This allowed us to take other relevant perspectives into account and to increase awareness of the instrument. Therefore CK interviewed five stakeholders with different cultural and religious backgrounds and relevant expertise on diversity and social justice issues in care from outside the healthcare organization. These experts were recruited through snowball sampling. Three were female and two male. Two were researchers, one was an intercultural philosopher, one the director of a youth care organization and one an advisor for health and wellbeing. Additionally, we discussed the instrument with the intercultural consultant of the healthcare organization, in a team meeting, during a meeting of healthcare professionals involved in CES support (CES network) and obtained feedback from the board of directors. All feedback was deliberated upon with the community of practice.

#### Phase 3

In phase three, CK tested the eight-step deliberation method articulated in the Diversity Compass in four additional contexts. Additional pilot testing helps to overcome potential issues within the core working group (community of practice) who, as they grew together over time, might have found it difficult to stay critical on the final version of the instrument [41]. Three of the pilot tests were done with diversity ambassadors, some of which had also participated in a focus group in phase one. One pilot test was done during an organization-wide symposium. CK used the eight steps to structure the dialogue on a specific situation that a participant had experienced and in which he or she was confronted with a diversity-related moral challenge. Also, the participants were given the opportunity to provide feedback on the seven recommendations that had previously been formulated by the community of practice and formed a core element of the tool. The pilot tests also served as a way to disseminate the instrument in practice by creating awareness among others about its existence.

As a result of each of these pilot tests final edits were made to the formulation of the dialogical recommendations and reflective questions. This led to the final version of the Diversity Compass. Once the content was finished, an external editor adjusted the language to increase readability for people with different literacy levels and language skills and an illustrator designed the final layout of the ‘Diversity Compass’ to make the document more attractive.

## Results

In this section we describe the outcome of the PD process: the Diversity Compass.^1^ We focus on its core elements. The Diversity Compass is a small booklet consisting of an introduction, seven conversation recommendations, an eight-step deliberation method, two practical examples that illustrate how the deliberation method can be used and information on support structures within the organization that can be approached by professionals who are experiencing diversity-related moral challenges.

The introduction of the Diversity Compass elaborates on our broad conceptualization of diversity, describes the aim of the Diversity Compass and acknowledges the need for ongoing dialogue about moral challenges related to diversity at different levels of the organization. In this section, we will present the seven recommendations (see Table 2), the deliberative method (see Table 3) and we will provide a short case discussion (see Table 4) to illustrate how the diversity compass can be used.

### Seven recommendations

One core element of the Diversity Compass are seven recommendations for engaging in dialogue about diversity-related moral challenges. They are based on the practical experiences and theoretical knowledge of the community of practice and the expert consultations.

The goal of the recommendations is to create awareness among healthcare professionals and managers about important preconditions for deliberating on a morally challenging situation related to diversity in a way that fosters openness and understanding of different (moral) perspectives and experiences. The recommendations also provide concrete reflective questions for dialogue and deliberation. They have been summarized and translated from Dutch to English in Table 2.

**Table 2:** The seven recommendations

**1. Be attentive to (your) feelings**
Differences can lead to emotions and feelings, for instance uneasiness. These tell you what you want, need and what is important to you. By being aware of and addressing your feelings, you can learn more about yourself and each other, also in a professional context. If you are honest and dare to be vulnerable, then others may also do so. Especially if you have a position in which you can set an example, for instance as a manager. Ask yourself: How do I feel in this situation? Why do I feel this way? How does the other person feel?
**2. Start with yourself. Recognise and test your own judgements**
Dealing with moral challenges related to diversity well starts with yourself. Everybody has assumptions and prejudices. These are often related to your background, for instance how you grew up. At work you are also not neutral. For a good conversation it is important to be aware of this. Ask yourself: What do I think about this? Where does my perspective come from? And are my assumptions actually true?
**3. Show earnest curiosity**
Not all people with the same cultural background or sexuality and gender identity are the same. Don’t categorise people. You don’t know what the other person really thinks or feels if you don’t ask them. Be curious. However, also be patient and listen actively. Ask yourself: what don’t I know yet? And ask the other person: what is important to you, and why? But be careful and respect personal boundaries. Not everybody wants to discuss their sexuality with colleagues. Also pay attention to your own boundaries when you feel like someone else is trespassing them
**4. Be respectful. Give the good example**
Although you may be different from someone else, all people are equal. You can show respect through the way you talk, your body language and by asking earnest questions. Treat the other like you want to be treated. If you have the feeling that someone is not treating you respectfully then make yourself known. Ask yourself and the other: are we respectful in the way we treat each other? And, if not: how can we change this?
**5. Engage in dialogue, not in discussion!**
The goal of a good conversation about diversity is not to convince each other, but to understand each other better. The way you communicate is key. Be open to each other and accept differences. Try to put yourself in their shoes and listen. Accept that the outcome of a conversation can also be that you have different perspectives. Figure out together how you can take all perspectives into account in the way you provide good care
**6. Support each other**
Offering and getting support can help with having difficult conversations about diversity. Support can mean different things. For instance, you can ask a colleague who speaks the native language of a patient to help you when providing care. Or, if you feel alone and isolated, you can ask a colleague you trust to support you before or during a difficult conversation with your team. Also offer support to others that need it. Ask yourself: who can help me in this situation, or whom can I help?
**7. Ensure safety and trust**
(Psychological) safety and trust are important to have good conversations. Especially in situations where hierarchy or power differences play a role or when you are afraid of the reactions of others. Dare to speak out if you feel unsafe and watch out for each other. Ask yourself and each other: what can I/we do to ensure psychological safety in this conversation? What do we need so that we can really be open and vulnerable?

#### The eight-step deliberation method

In addition to the seven recommendations, we also developed a short, structured deliberation method consisting of eight steps. The method can be used to prepare for a challenging conversation with a colleague or manager or to structure individual or group reflections among healthcare professionals on specific situations that have to do with moral challenges related to diversity. The aim of the method is to prepare for and to dialogically address concrete, diversity-related moral challenges in order to gain insight into the moral values and perspectives of different stakeholders, to facilitate moral learning and to eventually engage in more diversity-responsive care and communication. This method is shown in Table 3 and has been translated from Dutch to English by the first author.

**Table 3:** Structured deliberation method

1. What is the situation?
2. What are your feelings about the situation? What is so difficult about this situation?
3. What is your specific question?
4. What are the feelings of the others? (Your conversation partner(s) and other stakeholders involved in this conversation, for instance your patient, colleague, or manager. Ask them for their feelings, or try to put yourself in their shoes if they are not present during the dialogue)
5. What is important to you? How does your social background influence what you find important? Where and how you grew up, your age, cultural background, spiritual beliefs, sexuality and gender identity, etc
6. What is important to the others? Also inquire and reflect on their background
7. What do you learn from this exploration?
8. What will you do? If you go through these steps with others, first ask them what they would do in your place

A necessary prerequisite for engaging in dialogue using this method, is that the seven recommendations are upheld. This also means that the conversation must occur in a safe and respectful way in order to create a setting in which moral learning and open dialogue can take place, prejudices and blind spots can be reflected on openly and to refrain from perpetuating discrimination, racism, sexism or other forms of injustice, despite best intentions. The method also implicitly incorporates some of the seven recommendations. For instance, question two explicitly asks those engaging in dialogue about feelings and emotions and what these say about what is considered difficult or important to the case presenter and other participants (see also recommendation 1). Additionally, the method reflects a broader conceptualization of diversity as related to someone’s social background that consists of different (intersectional) aspects including where and how someone grew up, age, cultural, spiritual background and sexuality.

Based on the preferences of the participants during the PD process, the deliberation method can be used flexibly to discuss a specific case example. This means that there is no specific time frame for engaging in dialogue with this method, and that it may be used individually, with one conversation partner or in groups. This flexibility was a key condition proposed by participants so that the instrument may be used in different care contexts and circumstances. Further, the deliberation can occur as part of existing meetings like multidisciplinary case discussions, team meetings or individual conversations between managers and professionals.

In case of group dialogues (with at least three people), one person within the group is the denominated case presenter and another person acts as the facilitator. The facilitator is responsible for guiding the reflection process by following the question-based deliberation method and ensuring equal participation of all participants. All participants are jointly responsible for adhering to the seven recommendations described earlier. After the deliberation method is used, the participants evaluate the reflection process together.

We shortly illustrate how the dialogical conversation method in the Diversity Compass can be used to reflect on a morally challenging situation that is related to a diversity, or social justice issue in Table 4. The following case is based on a dialogue carried out in the course of this study with the help of the Diversity Compass but has been adjusted and summarized for confidentiality reasons. The names used below are pseudonyms.

**Table 4:** Case discussion

Here, we provide a case discussion loosely based on a case provided by a participant in this study. In this example a healthcare professional (the case presenter, Alex) reflects on a diversity-related moral challenge with one other healthcare professional (Ezra) by means of the deliberation structure proposed in the Diversity Compass. Before engaging in dialogue, they critically examined the seven recommendations. The diversity-related moral challenge at hand concerns Alex reflecting on how to approach his colleagues whom he had overheard joking about a transgender patient who is transitioning.
**1. What is the situation?**
Alex: Since recently we care for a transgender patient who is transitioning. Last week two colleagues of mine left their room and I overheard them making a joke about this patient’s body
**2. Wat are your feelings about the situation? What is so difficult about this situation?**
Alex: I think that this is disrespectful! I get angry and disappointed at my colleagues for talking this way. I also don’t find this professional behavior. What I find particularly difficult is how I ought to talk to my colleagues about what I heard. I think that healthcare professionals should not discriminate or laugh about others based on their gender identity. I want to have a serious and open conversation with them, but am not sure how I can do that in a good and constructive way. I am also a bit scared about damaging my relationship with my colleagues if I address this situation
**3. What is your specific question?**
Alex: How can I address this situation and my feelings about their behavior with my colleagues in a good way?
**4. What are the feelings of the others? (Your conversation partner(s) and other stakeholders involved in this conversation, for instance your patient, colleague or manager. Ask them for their feelings, or try to put yourself in their shoes if they are not present during the dialogue)**
Alex: Although the patient was not present in this situation, I think that they would be very hurt by my colleagues’ comments. I also got the impression that my colleagues did not really understand why they were transitioning which they masked with humor and laughing. Regarding my manager, I am not sure what her feelings would be about this situation. We never really address situations like that in our team Ezra: This is so terrible to hear. My first reaction is that the comments of the colleagues are unprofessional and unsuitable, this situation frustrates me. I can also only imagine how denigrating and painful it may be for the patient to be talked about in this way
**5. What is important to you? How does your social background influence what you find important? Where and how you grew up, your age, cultural background, spiritual beliefs,** sexuality and gender identity**, etc**
Alex: I think that this situation is so difficult for me because I grew up with the notion that you have to be open towards each other and treat each other with respect. My parents always said: treat others as you want to be treated. From personal experience I know what it is like to be ridiculed or bullied because of who you are. It was only when I got older and moved to a larger city that I really felt accepted. That is why it is so important to me that all my patients are and feel respected and that my colleagues do not make these kinds of discriminatory ‘jokes’. It is difficult for me to work with people that talk in this way. I really want my colleagues to reflect on their behavior
**6. What is important to the others? Also inquire and reflect on their background**
Alex: I think that it is important to the patient that they are respected for who they are. And that they receive good and professional care without being discriminated against. And my colleagues probably want to openly share their opinion with each other. Actually, I am curious to know what they really find important, what inclined them to make this joke and what the perspective of my manager is on this situation Ezra: I am wondering whether your colleagues are actually aware of their behavior and its consequences and if they know how difficult the transitioning process is and how much discrimination there is. In general, I think it is really important that the whole team makes clear and concrete agreements about what they perceive as a good way to talk about patients. And that discrimination is never okay. Having an open discussion in your team where you critically reflect on this and similar situations in the future could help with this, if it is done in a good way and facilitated by someone who makes sure that it is a safe and respectful conversation. I think that this may really contribute to better and more inclusive care
**7. What do you learn from this exploration?**
Alex: I realized that my colleagues might act like this based on discomfort and a lack of knowledge and insight into the situation. And they might not have thought about how hurtful their comments may be to the patient and to others. Most importantly, I learn that I really have to talk to my colleagues and manager about this
**8. What will you do? If you go through these steps with others, first ask them what they would do in your place**
Ezra: I would talk about this with your colleagues at a moment where there is enough time. You should also set the ground rules for the conversation by discussing the seven recommendations with them, before starting with the actual subject and really focus on your own perspective. You can also ask a colleague you trust to support you. Or your manager Alex: I will use this conversation method with my colleagues tomorrow. I think that colleague D may help me here. She is a good friend and has experience with guiding dialogues. She can help me when I am uncertain about what to say and will make the conversation feel safer. Through the dialogue I want to know more about my colleagues’ true motivation, to share my feelings and really reflect on the situation together
**Evaluation:**
Through this dialogue I realized that sometimes people may not be aware of the fact that they see someone else, or their behavior, as different or even inferior. These ‘blind spots’ are important to talk about so that you can treat each other respectfully – not only those that ‘are like you’. I will address this specific situation with my colleagues, and I also want to talk about social justice issues more regularly in my team. My manager may also help, for instance by making diversity and inclusion a regular topic in team meetings. I will also have a look if there are any people in my organization who can help with these conversations

## Discussion

Here, we will critically reflect on the development process and the content of the Diversity Compass. Additionally, we will provide some recommendations for future research and practice.

### The development process

First, we would like to reflect on the PD process that we used to develop the Diversity Compass. PD is based on the assumption that inclusion of stakeholders, and particularly engaging in co-creation together with end-users, is beneficial for the quality and feasibility of a new intervention [41, 43]. It has been argued before that participatory approaches are valuable for developing and evaluating (dialogical) CES instruments [36, 48]. Participation may also help with bridging the gap between research and practice [49], as well as with creating momentum, ownership of the intervention and a network of relations, which all increase the likelihood of attaining practical implementation and social impact [39, 40]. By engaging in dialogue with different stakeholders for a period of thirteen months in this study, we tried to facilitate more awareness of social injustices, different perspectives and experiences of diversity and inclusion and the importance of addressing diversity-related moral challenges regularly and at different levels throughout the healthcare organization.

Nonetheless, creating and executing a developmental process for a CES instrument that is congruent with the subject matter itself, i.e. diversity and social justice, is in itself a moral challenge. Especially when considering the extent to which this study fully achieved to be participatory, dialogical and inclusive. Although we tried to be as inclusive as possible in the different phases of the PD process, also by actively including and listening to the voices of several peer-experts and (former) patients, some perspectives were missing, such as that of informal caregivers. This is an important criticism related to social justice, specifically as dealing with the moral challenges that were discussed in this study and in the Diversity Compass itself, is strongly dependent on who has a seat at the table. Additionally, the exclusion of those with marginalized voices perpetuates epistemic injustices despite best intentions [39]. Therefore, we acknowledge that including additional and different stakeholders in the study could have offered insights that might have influenced the developmental process and outcome. Also, in future research, the perspectives of care-receivers have to be included actively when evaluating the deliberation process and outcomes of using the Diversity Compass.

Moreover, it is important to critically reflect on our own role and position as people and ethicists in facilitating the PD process that led to the Diversity Compass. We actively participated in the production of the instrument. Rather than being neutral observers, our own social location and normative background as people, ethicists and researchers influenced our interaction with the participants and the set-up and outcome of this study.

The team that facilitated the development of the Diversity Compass consisted of white, cisgendered women with different western European backgrounds and at different stages of their academic careers. The limited cultural and gender diversity in the composition of the research team may have contributed to researcher bias and can therefore be considered a limitation. While we hope to have mitigated potential blind spots and biases as much as possible by engaging in a co-creative process with individuals with various intersectional social identities and we actively sought feedback and support from professionals with different minoritized identity characteristics, we are aware that our personal background influenced the conception, process and outcome of this study.

Additionally, from a methodological and normative standpoint, our approach to doing ethics in healthcare is routed in philosophical pragmatism, hermeneutic ethics and discourse ethics and is based on the idea that moral learning takes place by deliberating on concrete experiences, contextual knowledge and by placing yourself in the shoes of others [25, 28, 31–33, 36]. This also means that content ought to be determined together and knowledge is dependent on the stakeholders, process and specific context in which reflection takes place [2]. This approach is different to other CES contexts and strongly defines the developmental process we engaged in here, i.e. by facilitating dialogue to develop a dialogical CES instrument. Other normative approaches to CES, likely would have led to a different process and tool.

Moreover, the study was financed by the healthcare organization itself. We were invited as ethicists and researchers by the diversity officer of the healthcare organization to develop a tool to promote diversity-responsiveness. Additionally, some of the participants were recruited from the personal network of this diversity officer, who was also a director of the organization, which likely lead to elements of bias in some of the group compositions. Both considerations raise questions regarding our own impartiality, independence and biases in the developmental and recruitment process that led to the Diversity Compass. The setting also creates the possibility of a conflict of interest: carrying out our study in a way that is fully in accordance with our academic and ethical bonafide way is possibly at odds with completing our assignment in a way that is satisfactory to the organization who issued it. What if the organization was not happy with the outcomes or tools? What if we needed more time and resources for our study? What mitigated the risk of biases and a conflict of interest, was that we discussed these issues upfront and stressed the importance of our own independence and freedom in shaping and conducting the study. The organization could agree on this. Before the start of the project, we wrote an elaborate research proposal including our planning, approach, (recruitment) methods and envisioned outcomes. This created a situation in which this risk was not fully eradicated, but at least expectations on both sides were managed from the start and we tried to address inherent biases as much as possible.

Furthermore, in we believe that a risk of a conflict of interest is immanent to practically all situations where clinical ethicists engage in studies together with healthcare organizations and professionals that seek to change practices [25]. Especially in participatory research, those with lived experience (participants) and external researchers engage and learn with each other in order to challenge traditionally asymmetric relationships and move towards an inclusive approach where they can create new ways of acting and knowing, together [39]. This presupposes interdependency and immersion of the researcher in practice, rather than a detached, independent and supposedly ‘unbiased’ researcher.

### The Diversity Compass

A vast body of evidence has shown that health care needs are unequally met across social communities, linking social inequalities to structural health disparities among different, minoritized patient populations [3, 8–12]. Additionally, there is unequal gender and minority representation in the healthcare workforce, especially among physicians [13]. This demonstrates disparities in recruitment and is another challenge in achieving equity in organizations [12, 14] and promoting cultural competence in health and care [15]. Dealing with these inequities requires a systemic approach. However, supporting healthcare professionals to recognize and attend to patients’, colleagues’ and their own social identities that can underlie their moral perspectives, may *contribute* to more inclusive and just work environments, and help professionals with providing good care for all.

The objective of the Diversity Compass is to support healthcare professionals to reflect on and address moral challenges related to intersectional aspects of diversity and social justice issues that they experience in daily practice in a structured, dialogical way. The goal is to stimulate moral awareness, learning and dialogical competences among healthcare professionals and help them with delivering diversity-responsive care by actively considering their own and others’ social identities. In a way, the process that led to the development of the Compass may thus be seen as part of the objective and outcome of this study. This is because we facilitated dialogue with different stakeholders about their perspectives on and experiences with diversity and social justice, thereby trying to create awareness and facilitating reflection on those very subjects throughout this study.

Furthermore, on purpose, the Diversity Compass provides no answers or concrete moral judgments regarding the right thing to do in a specific situation in which someone experiences a diversity-related moral challenge. Rather it consists of seven recommendations and a dialogical reflection method. This is different to the way diversity is often approached in bioethics, i.e. by mainly addressing theoretical concerns when balancing diversity with other ethical principles, or reflecting on moral pluralism and cultural diversity in general [50, 51]. However, this approach is similar to other dialogical methods in CES and ethics education [27, 30, 31, 36, 52].

Some benefits of the Diversity Compass are that it was designed to respond to the needs of end-users, can be used flexibly and independently by different care professionals and, most importantly, that it is specifically tailored to addressing diversity-related moral challenges in healthcare practice. Given increasing social pluralism, diversity in social identities and existing disparities in care access and practices [3, 8–12], it is necessary to provide support with facilitating more inclusive and diversity-responsive care and work environments [16, 24] and to do so by taking an intersectional approach [19, 20, 53]. The Diversity Compass was designed to respond to this need. It is a practical, low-threshold instrument that can be used in different healthcare contexts and is responsive to the inherent complexity of engaging in dialogue on various moral perspectives between different, minoritized and majoritized stakeholders.

However, despite these benefits, reflecting on diversity-related moral challenges requires modesty and vigilance. It is important to watch out for problematic stereotyping and to address biases, blind spots and potential pitfalls, also regarding the content of the Diversity Compass. The Diversity Compass is a dialogical instrument. However, dialogical practice does not guarantee that the process of the dialogue or its outcome is inherently just or morally good, and *actually* does what it tries to accomplish. Therefore, a key issue regarding the Diversity Compass refers to the question how and whether this instrument can *ensure* an inclusive and diversity-responsive reflection process and deliberative outcome.

This is particularly concerning as others have argued that healthcare professionals may specifically refer to cultural differences between themselves, patients, families, or populations, as a way to mask their own ethical uncertainties, biases, racism or moral distress [16, 21]. Also, it has been argued that normalization of or indifference towards social hierarchies, privileges and disadvantages remains a challenge in Dutch healthcare organizations that particularly impact minoritized healthcare professionals [54]. When considering the dialogical nature of the Diversity compass this may, for instance, mean that professionals who discuss a case could end up over-problematizing certain aspects of diversity or someone’s social identity as the reason for why they experience a situation as morally troublesome, rather than detecting systemic social justice issues or gaining insight into personal prejudices instead of blaming the Other. Personal and systemic blind spots and biases may lead to a lack of awareness of social inequities [22, 23]. These considerations signify a key challenge for using a dialogical intervention like the Diversity Compass, where the particularities (and perceptions of the quality and inclusiveness) of the process and outcome is dependent on those participating in the deliberation.

A cornerstone of the Diversity Compass are the seven recommendations which we consider necessary to uphold when engaging in the actual deliberation method in order to facilitate a safe environment for dialogue to occur. This means that being attentive to feelings, recognizing and reflecting on personal prejudices, showing earnest curiosity, respect, the will to engage in dialogue, to support each other and facilitate safety and trust as much as possible, are central to creating the necessary conditions in which the reflection method should be used. Organizations and professionals have a shared responsibility to facilitate an inclusive space in which non-hierarchical dialogue can occur, power relationships can be addressed and a moral judgment can be formulated that actually promotes diversity-responsive care and equity within organizations, rather than, for instance, encouraging stereotypes, racism or unsafe environments for minoritized others. However, there is no definitive certainty that the Diversity Compass will always be used to this end.

A final issue relates to the implementation of the Diversity Compass and to the question to what extent it *actually* contributes to more inclusion and diversity-responsiveness in care and work environments. Sara Ahmed has rightly warned for the ‘non-performativity’ of doing diversity in organizations: diversity documents are often mere paper trails with no, or little actual effect on transforming institutions or facilitating more equality [55]. Although Ahmed focusses on the (non)effect of policies rather than on ethics support instruments, this concern also applies here. Others have also argued that employing CES instruments structurally, is key to ensure that attention for the ethical dimension of care is meaningful and ongoing [34]. Moreover, a single instrument like the Diversity Compass alone is not sufficient to address diversity-related moral challenges and facilitate social justice and organizational change. Rather, different organizational strategies, policies, ethics and diversity training and interventions ought to be implemented and dialogue should occur regularly at all levels of healthcare organizations in order to create an environment that is safe and inclusive of all [56] and where diversity-responsiveness becomes the norm. Working toward social justice requires a structural and systemic approach in organizations [16, 26].

### Recommendations

The Diversity Compass is designed with and for a particular healthcare organization based on the wishes and needs of the healthcare professionals that participated in its development. It is currently embedded within a diversity toolbox that is used by professionals in the diversity ambassador network that were key contributors to the development of the instrument, in their daily work environments. However, further research is required on the feasibility, effectiveness and implementation of the Diversity Compass. This includes studying if, where and how (frequently) the dialogical CES instrument is being used and whether it *actually* contributes to moral learning on diversity and social justice issues and to more inclusive care and work environments in practice.

Additionally, future research is necessary to critically reflect on and examine how diversity issues can be addressed in a recurring and systematic way and at different levels of healthcare organizations through dialogical CES, including in policy, care strategies and recruitment, in order to facilitate sustainable change towards increasing social justice in healthcare organizations. It is likely that, to achieve desirable and long-term change, healthcare organizations should structurally implement and integrate the Diversity Compass into existing team meetings at different levels of the organization. This also requires training healthcare professionals and managers to use the tool in the way it is intended, i.e. by adhering to dialogical principles.

In order to make healthcare more diversity-responsive and just with the help of CES, an instrument like the Diversity Compass is not enough. It cannot stand on its own. Rather, we believe that facilitating diversity-responsiveness and equity requires a comprehensive approach that combines different interventions, including suitable organizational policies and strategies, training, ethics and diversity education and continuous reflection and dialogue at all levels of an organization. This also means that clinical ethicists themselves should be trained to engage in more diversity-responsive practices in the CES they provide and the reflection tools they develop. Furthermore, an inclusive organizational culture and ethical climate – in which dialogue about social justice is valued, and in which moral questions related to diversity are attended to and approached with care and accountability – are necessary prerequisites to safely reflect on diversity-related moral challenges in systems where hierarchies and power relationships exist. Thus, the Diversity Compass should be part of a structural endeavor to promote diversity-responsiveness and social justice in healthcare organizations.

## Conclusions

In this paper, we described the development and content of the Diversity Compass, i.e. a CES instrument that seeks to support healthcare professionals in dealing with diversity-related moral challenges in practice. The Diversity Compass is a low-threshold dialogical ethics support instrument that aims to encourage awareness, moral learning, mutual understanding, and critical reflection on diversity and social justice issues to, eventually, contribute to more inclusive and diversity-responsive care and work environments. By means of a PD design, we sought to foster a joint and inclusive learning process among various stakeholders and end-users in order to experiential knowledge in the Diversity Compass, and to encourage ownership and implementation of this instrument in daily practice.

However, while facilitating dialogical reflection may contribute to a more inclusive workplace in which professionals are able to deal better with diversity-related moral challenges, an ethics support instrument such as the Diversity Compass alone is insufficient to warrant a continuous awareness and actual change. If the use of the Diversity Compass is not embedded in a larger and structural endeavor to deal well with challenges concerning diversity and social injustices, it may have no or little social impact and, in a worst-case scenario, it might even perpetuate stereotypes or blindness to systemic injustices such as discrimination and exclusion in the workplace and in health care provision. Rather, fostering equity, inclusion and social justice is an ongoing process that requires a multi-faceted approach as well as continuous attention and reflection among different stakeholders at all levels of the organization.

### Supplementary Information

**Additional file 1. **Semi-structured interview guide: focus groups.

## Data Availability

For privacy and confidentiality reasons, the qualitative data on what was discussed during the interviews, focus groups and community of practice is not publicly available. Any requests about data availability should be directed to the corresponding author.

## Abbreviations

CES: Clinical Ethics Support
PD: Participatory Development

## Footnotes

1 The Diversity Compass can be downloaded here: https://www.xendens.nl/p/46/diensten/het-diversiteitskompas-goed-omgaan-met-verschillen
