# Supplementary material for: The Diversity Compass: a clinical ethics support instrument for dialogues on diversity in healthcare organizations
Source: BMC Med Ethics. 2024 Jan 3;25:4. doi: 10.1186/s12910-023-00992-z (PMC10765795; doi:10.1186/s12910-023-00992-z)
Supplement: Supplementary file 1 — Additional file 1. Semi-structured interview guide: focus groups. [file 12910_2023_992_MOESM1_ESM.docx]

**Semi-structured interview guide: Focus groups**

1. How do you define diversity? Which aspects of diversity are especially important in healthcare?
2. Which experiences have you had with dealing with diversity (well) in daily practice?
   1. Wat is a good experience you had (best practice)?
   2. What is a negative experience that you personally encountered?
3. Have you experienced any moral challenges related to diversity and inclusion in your respective work environments? Can you provide examples?
   1. What are underlying emotions, norms and values that are important to you regarding these challenges?
4. Which support have you received from your manager or the healthcare organization you work for in dealing with diversity and the challenges you encountered well?
   1. What is needed (at the individual, group, management and organizational level) to address diversity well in practice? What is going well, what could be better?
5. What does a good ethics support instrument have to look like to support you with dealing with diversity-related moral challenges? (Content and form)
   1. What do you need to use such an instrument in practice?
   2. Are there any particular moral challenges or themes that the instrument should consider in regard of diversity and inclusion?
6. What should the ethics support instrument not look like?
7. Do you have any additional questions, ideas or insights you would like to share?
